# Castleman’s disease mimicked melanoma metastasis in the mesentery – A case report

**DOI:** 10.1016/j.ijscr.2020.02.001

**Published:** 2020-02-06

**Authors:** K.R. Liedtke, N. Waldburger, A.S. Glitsch, A. Schreiber

**Affiliations:** a Department of General, Visceral, Thoracic and Vascular Surgery, University of Greifswald, Sauerbruchstraße, 17475 Greifswald, Germany; b Institute of Pathology, University of Greifswald, Friedrich-Loeffler-Straße 23e, 17475 Greifswald, Germany

**Keywords:** Case report, Castleman’s disease, Intraabdominal tumor, Lymph node swelling, Mesenteric lymphadenopathy

## Abstract

**Introduction:**

Castleman’s disease (CD) is a rare and mainly asymptomatic cause of lymph node swelling. Often it is unicentric and located in the mediastinum. Due to rarity of the disease as well as a lack of symptoms, diagnosis proves to be challenging, especially when CD affects another region.

**Presentation of case:**

A 51-year old male underwent resection of a malignant melanoma. Further staging revealed an unclear abdominal mass located in the mesentery with close contact to small intestine. Under the assumption of metastasis, complete tumor removal including intestine resection and anastomosis was performed. Both, operation and postoperative phase proved uncomplicated. Surprisingly, however, histology revealed a benign lymphoproliferative disorder, CD.

**Discussion:**

There are several differential diagnoses for abdominal soft tissue tumor, such as: gastrointestinal stromal tumor, sarcoma, lymphoma, or metastasis. In reference to the resected melanoma described above, metastasis was assumed with subsequent oncological resection. Both, the reliable detection of CD as well as the exclusion of malignant disease (*e.g.* lymphoma) can only be achieved through pathology, in that specific tests fail yet to exist. The etiology of CD remains barely understood and based upon few cases reported complete surgical resection is recommended. However, the common form is meant to be benign.

**Conclusion:**

The potential diagnosis of CD should be made more common to surgeons, especially in completely asymptomatic patients and non-superficial lesions, whereby close follow-up examination might be offered to patients.

## Highlights

• Castleman’s disease (CD) is a rare finding and diagnosis is very challenging.
• Intraabdominal localization is uncommon and can pretend malignancy.
• Misinterpretation might lead to surgical overtreatment of asymptomatic patients.
• Close follow-up examination might help to distinguish between malignancy and CD.

## 1 Introduction

Castleman’s disease (CD), also known as angiofollicular lymph node hyperplasia, is a rare, per se benign lymphoproliferative disorder. This disease was first systematically described in 1956 by and later named after Benjamin Castleman [1]. Frequently, the patients present themselves completely asymptomatically, or with unclear swelling of the lymph nodes. Diagnosis is correspondingly a chance finding [2,3]. Nevertheless, this detection may have relevant consequences for the patient: on the one hand in the presence of a multicentric type of CD, which is associated with a significantly reduced prognosis, on the other hand, the disease may be misdiagnosed as a malignancy with consecutive overtreatment. Recently, such a case was uncovered and underwent subsequent treatment at the University Hospital, Greifswald, Germany. In this instance, a unicentric CD was misinterpreted as a distant metastasis of a recently resected malignant melanoma. This work is a report of this experience in accordance with the SCARE criteria [4].

## 2 Case report

**Fig. 1:**
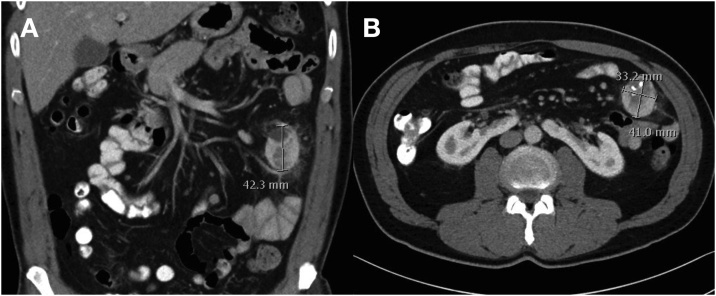
CT scan with suspicious finding at the mesentery. In the mesentery with close contact to the small intestine, we detected a both, cystic and calcified area with a size of 4.2 × 4.1 × 3.3 cm. In addition, strong contrast enhancement and imbibed perifocal adipose tissue, as well as suspicious swelling of the perifocal lymph nodes was described. As a secondary finding, a horseshoe kidney resulted, otherwise no interesting findings. Images in coronary (**A**) and axial alignment (**B**). CT scan is enhanced by oral, rectal and intravenous applicated contrast agent (iodine based).

51-year old, male patient with a basically insignificant medical history except for arterial hypertension, a recent meniscus surgery, and an appendectomy in childhood was first referred to the Department of Dermatology with a highly suspicious for melanoma mass in the transition from the back to the right gluteus maximus. Post resection, subsequent histopathological workup revealed a superficially spreading, malignant melanoma with a diameter of 3.5 cm and a depth of 3.5 mm. The tumor formula was: pT3 L0 V0 R0. However, due to an only minimally tumor-free margin (approx. 1 mm), yet another surgical resection including puncture of the scintigraphic (28 MBq ^99m^Tc-Nanokoll) defined sentinel lymph node was performed. This subsequent resection revealed the necessary 2 cm safety margin. The lymph node was histopathologically free of malignancy. As part of the extended staging, a whole-body CT was undertaken. Cerebral or pulmonary tumor manifestations could not be detected, however, an unclear lesion of 4.2 × 4.1 × 3.3 cm in size was observed intraabdominally in the left middle abdomen (Fig. 1). This exhibited both cystic as well as calcified areas, positive enhancement and the perifocal adipose tissue was imbibed. The lymph nodes along the Aa. iliacae demonstrated moderate, suspicious swelling (up to 15 mm shortest axial diameter). Further malignancy-related findings were not observable. From a radiological point of view, a metastasis of the known melanoma or a primary tumor of the mesentery (*e.g.* neuroendocrine tumor, gastrointestinal stromal tumor) appeared possible, as well as a benign lesion such as, for example, sclerosing mesenteritis. The lesion was inaccessible to a CT-assisted puncture, therefore after endoscopic tumor exclusion (*i.e.*, gastroscopy, colonoscopy), the decision for surgical exploration was made. Post sparing median upper abdominal laparotomy, the above-described tumor was located within the mesentery, very close to the small intestine and approximately 100 cm post ligament of Treitz. The tumor and the surrounding mesentery presented palpably solid and suggestive of malignancy. A small intestine resection was carried out along with the mesentery to the aorta, so that ultimately 33 cm small intestine and an 18.5 × 11.5 × 3.7 cm large piece of mesentery were resected. The anastomosis was uncomplicated, and the surgery was completed without any complications. The postoperative phase was also uncomplicated, so that we were able to discharge the patient to outpatient follow-up on the fifth postoperative day.

**Fig. 2:**
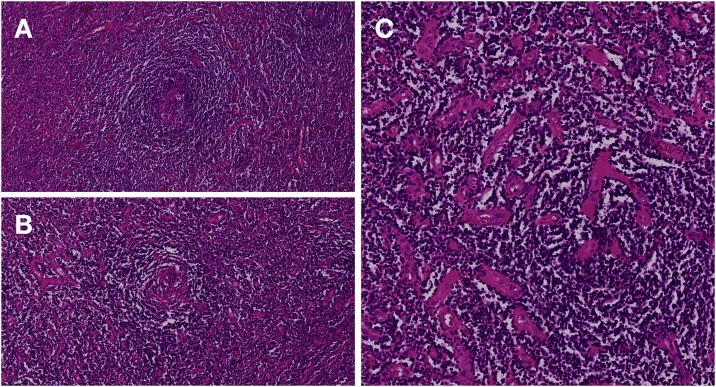
The suspicious lymph node showed typical signs of CD. Thickened mantle zones with concentric layers of small lymphocytes (onion skin appearance, **A**). Small vessels are penetrating the atretic germinal center creating a lollipop-like appearance (lollipop follicel, **B**). Hypervascular interfollicular tissue consisting of vascular proliferation with hyalinized walls (**C**). All images stained for hematoxylin and eosin (H&E) with 12x magnification.

Histopathological analysis revealed angiofollicular hyperplasia (*i.e.* Castleman disease, Fig. 2). The lymph node architecture itself was conserved and no evidence of infiltrates of malignant melanoma or any other malignancy was found. The other lymph nodes and the small intestine were completely unremarkable.

## 3 Discussion

CD is a rare diagnosis with little clinical expertise. As first described by Benjamin Castleman, the most common site of manifestation is suspected to be the mediastinum. The disease, however, can affect any lymph node, though rarely occurring within extra lymphatic tissue [5,6]. Interestingly, for the majority of cases (40%) observed by Yu et al., CD was discovered within the abdominal cavity without further specification [7]. In a review of 195 CD cases situated within the abdominal cavity, 63% were localized within the retroperitoneum, 14% in the pelvis (*e.g.*, the adnexa or even subperitoneal) and the remaining 23% were discovered intraperitoneal, particularly within the mesentery [5].

The etiology of this disease remains poorly understood. From a clinical point of view, two variations are distinguishable: the unicentric form, which usually affects only one lymph node or at the most one lymph node region, and the multicentric spread. The latter is often coincident with the human herpesvirus 8 (HHV-8), but there are also idiopathic forms. Unicentric CD is generally not associated with HHV-8 and is usually characterized pathologically as a hyaline-vascular type, whereas the variant of the so-called plasma cell type or a mix type is less common [8]. This distinction is crucial, especially with regard to the prognosis. *Talat et Schulte* advocate a classification of the disease according to clinical-pathological findings in which the unicentric, hyaline-vascular CD, as in the presented case, is classified as class I with a 3-year disease-free survival of 92.5%. [9]. This underlines the inherent benign nature of this disease. There is, however, evidence that this variant is associated with a higher incidence of Hodgkin's lymphoma [10,11]. As ‘curative’ treatment, total surgical excision represents the therapy of choice in the case of unicentric CD [6].

The greatest challenge for the physician, however, is usually not the correct choice of therapy, but instead to initially establish a correct diagnosis. Due to the rarity of the disease, it is seldomly considered in the differential diagnosis. Additionally, the symptoms are nonspecific and lymph node swelling is usually a chance finding [12]. The signs are also unspecific in CT morphology: in most cases, the tumors are sharply circumscribed and hypervascularized, they may demonstrate homogeneous or heterogeneous intense enhancement following administration of contrast agent and show calcifications in about 10–30% of the cases [13,14]. In particular, the distinction of a malignancy (*e.g.*, lymphoma, sarcoma) or a lymph node metastasis is therefore challenging [15]. Information obtained from fine needle biopsy often results in an inconclusive diagnosis [16].

Intraoperatively, the CT finding of a large, inhomogeneous mass in the mesentery was confirmed and a complete oncological resection was performed. However, pathology revealed CD of a hyaline-vascular type, which are commonly considered to be benign. Due to the temporal correlation with the malignant melanoma, as well as the nonspecific presentation of the lesion on CT, a metastasis was assumed. Accordingly, oncological resection with partial resection of the adjacent small intestine was performed. Frozen section analysis was not performed, in that it might have rebutted the idea of melanoma. However, it seems very unlikely, that a lymphoma could have been precluded in that way. Finally, from a surgical point of view, the operation was carried out to the best of our knowledge. In retrospect, a close follow-up could have revealed the benign nature of the disease. Nonetheless, a bowel-preserving resection would have been almost impossible due to the close contact with the pathologic lymph node.

## 4 Conclusion

Diagnosis of CD is challenging and is often discovered by chance. An awareness of this benign disease in the instance of asymptomatic, isolated lymph region swelling may lead to the development of a correct diagnosis. In most cases surgical resection appears to be therapy of choice, as it is relatively uncomplicated and allows certain diagnosis by histopathology.

## Declaration of Competing Interest

None.

## Funding

This research did not receive any specific grant from funding agencies in the public, commercial, or not-for-profit sectors.

## Provenance and peer review

Not commissioned, externally peer-reviewed.

## Ethical approval

This study is exempt from ethical approval

## Consent

Written informed consent was obtained from the patient for publication of this case report and accompanying images. A copy of the written consent is available for review by the Editor-in-Chief of this journal on request.

## Author contribution

Kim R. Liedtke, MD, BSc: wrote the draft for case report, carried out the literature search, and wrote up the literature review.

Nina Waldburger, MD: performed the histopathological preparation and analysis, edited the draft.

Anne S. Glitsch, MD: assisted the surgery, edited the draft.

André Schreiber, MD: planned and performed the surgery, edited the draft, and helped to analyze the literature review results.

## Registration of research studies

This retrospective case report is not registered in a public database, yet

## Guarantor

Kim R. Liedtke, MD, BSc; André Schreiber, M.D.
